# PD1/PD-L1 immune checkpoint as a potential target for preventing brain tumor progression

**DOI:** 10.1007/s00262-021-03130-z

**Published:** 2022-01-29

**Authors:** A. Filippone, M. Lanza, D. Mannino, G. Raciti, C. Colarossi, D. Sciacca, S. Cuzzocrea, I. Paterniti

**Affiliations:** 1 Department of Chemical, Biological, Pharmaceutical and Environmental Sciences, University of Messina, viale Ferdinando Stagno D’Alcontres, 31, 98166 Messina, Italy; 2 IOM Ricerca Srl, via Penninazzo 11, 95029 Catania, Italy; 3 Instituto Oncologico del Mediterraneo, via Penninazzo 7, 95029 Catania, Italy

**Keywords:** PD/PDL-1 pathway, Immune system, CNS, Brain tumor, Immunotherapy

## Abstract

Programmed death-1 (PD-1) is a cell surface receptor that functions as a T cell checkpoint and plays a central role in regulating T cell collapse. The binding of PD-1 to its ligand programmed death-ligand 1 (PD-L1) activates downstream signaling pathways and inhibits T cell activation in the perspective of immune system mechanism and regulation in tumor progression. It is well reported that tumors adopt certain immune-checkpoint pathways as a mechanism of resistance against immune cells such as T cells that are specific for tumor antigens. Indeed, the PD-1/PD-L1 pathway controls the induction and maintenance of immune tolerance within the tumor microenvironment. Thus, the PD-1/PD-L1 checkpoint regulation appears to be of extreme importance as well as the immunotherapy targeting that via and the using of PD-1/PD-L1 inhibitors that have changed the scenario of brain cancer treatment and survival. Here, we review the mechanism of action of PD-1 and PD-L1, the PD/PDL-1 signaling pathway involved in the progression of brain tumors, and its application as cancer immunotherapy counteracting tumor escape in central nervous system.

## Introduction

Mammals have evolved complex immune strategies to contrast foreign pathogens and to preserve corporal health. Adaptive and innate immunity are the two crucial elements of the immune response. Lymphocytes B and T carry out both classes of responses, B cells activation drives immunoglobulins secretion neutralizing the host, while T cell-mediated response kills the virus-infected cells and produces signal molecules that activate macrophages to destroy the invading microbes that they may have already phagocytosed [1]. Among all immune regulators, some checkpoints represent an achievable target for mediating the immunosuppressive effects of varied malignancies. For instance, immune checkpoint’s inhibitory receptors such as programmed cell death protein 1 (PD-1) and its ligand programmed cell death ligand 1 [(PD-L1) also known as B7-H1], give rise to activation of immunosuppressive signaling pathways. PD-1/PD-L1 pathway controls the induction and maintenance of immune tolerance within the tumor microenvironment (TME). Particularly, PD-1 and PD-L1 are types I transmembrane proteins that belong to the immunoglobulin (Ig) category constituted by an Ig-V-like extracellular domain, a transmembrane domain, and a cytoplasmic domain [2]. PD-1/PD-L1 axis can be moderated by varied signals in cancer cells and, can act itself through regulation of PI3K/AKT, MAPK, JAK/STAT, and NF-κB pathways, critically involved in tumorigenesis processes [3]. Indeed, emerging findings highlighted that the functional inhibition of tumor progression and the high cancer cell proliferation by PD/PD-L1 overproduction, facilitate downstream activation and expression of involved molecules into tumor cell apoptosis [4]. In glioblastoma (GBM) cells, PD-1 ligands are mainly expressed as also reported in biopsies, hence the PD-1L impediment binding to its receptor PD-1 has been demonstrated to induce an immune escape mechanism validating that PD-1/PD-L1 inhibited could be a target for cancer immunotherapy of different tumor types [5]. Over the recent decade, immunotherapies aiming at PD-1/PD-L1 axis modification have obtained a series of remarkable discoveries in prognosis improvement of arduously to-treat solid tumors and are going to enter into the clinical practice of brain tumors. Early or late blockade of PD/PD-L1 checkpoint in association with potent T cell immunosuppressors has been demonstrated to neutralize T-cells (i.e. CD8+, CD8+, and CD44 +) subset and diffusion in glioma bearing-mice brain, lymph nodes, and spleens [6], presenting a hopeful treatment for patients with GBM. Though, the clinical efficacy of the PD-1/PD-L1 checkpoint blockade in brain tumors is still debated. All the current studies reported only the molecular signaling influence thus, the present review will discuss the PD/PD-L1 regulation in various types of brain tumors especially in brain glioma based on the blockade of this key immune system checkpoint. In this perspective, exploring effective targets and combination therapies to improve the clinical response of PD-1/PD-L1 checkpoint blockade is needed.

## Cellular signaling of innate and adaptative immune system

**Fig. 1:**
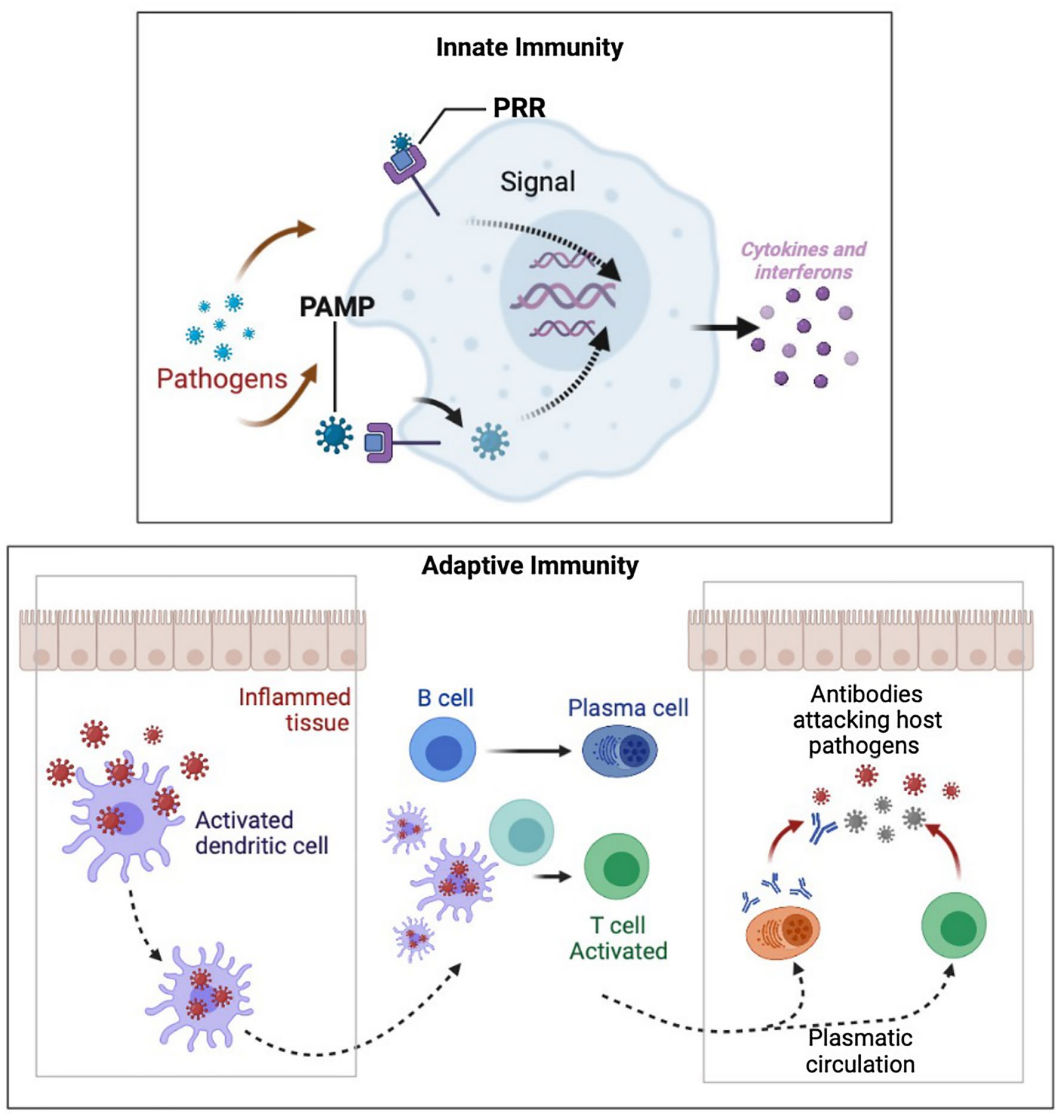
Innate and adaptive immune system cellular mechanisms

In a large measure, three typical extrinsic tumor-suppressor mechanisms have been acknowledged by which cells and own tissues sense cancerous cells existence. All these can belong to a unique circle of mechanisms that prevent cancer cells from invading and spreading to other tissues in the host: specific trophic signals diffusion in the cellular microenvironment, genes control of cells proliferation and differentiation, and tumor-suppressor mechanism involve the limitation of transformation or tumor cell growth by effector leukocytes of the immune system. De facto, immune cells have been found to play key roles for processes extending from embryogenesis to tumor clearance to host defense against pathogens. Cellular responses toward stimuli they receive emerge from interactions among proteins, and lipids mediated through specific binding sites. The immune system is the first to be activated following a new infection or pathogen entry stimulating immune players that are efficiently able to combat the pathogens [7]. It has been typically classified into innate immune response, having a rapid nonspecific and immunological memory, and T/B cell-dependent adaptive immune response, specifically slow and possessing solid immunological memory. First, is featured by recruitment and activation of neutrophils leucocytes colony at the site of infection releasing cytokines from activated macrophages in order to eradicate pathogens. The interactions allowing the innate response resolution, such as phagocytosis, and complement-mediated lysis, require the recognition of host molecules by cellular receptors on the surface of the immune cells. On cells of this type surface, pattern-recognition receptors (PRRs) (Fig. 1), of which the family of Toll-like receptors (TLRs) has been studied most extensively, are present at the cell surface and recognize distinctive pathogen-associated molecular patterns (PAMPs) (Fig. 1) identified as “nonself” host. During immunization, an antigen and a PAMP are generally mixed together and thus would not be perceived as having a common (microbial) origin unless both end up in the same endosome. This normally would require a large excess of PAMP over what is minimally required for activation of DCs. Specifically, the cellular components of innate immunity involve antigen-presenting DCs, phagocytic macrophages and granulocytes, cytotoxic natural killer (NK) cells, and γδ T lymphocytes. Cell types as macrophages, DCs, and B lymphocytes express TLRs, divided on the basis of the identification of nucleic acids (TLR3, TLR7, TLR8, and TLR9), and lipids (TLR1, TLR2, TLR4, and TLR6) [8]. Interestingly, some TLRs possess the ability to recognize structurally and biochemically unrelated ligands, as TLR4 to recognize such divergent structures as lipopolysaccharide (LPS), and cellular heat shock proteins (HSPs) [9]. In this perspective, the rationale for the PD-1/PD-L1 axis inhibition for adjuvant purposes is in the concept of “a high immunological memory” involved in glioma diffusion, starting from the early phases of tumorigenesis and through a number of different clues from adaptative immune system involvement [10]. The protection offered by innate cells includes regulatory mechanisms that contribute to the inclining of the derived adaptive immune response. It is well recognized that interferon-γ (IFN-γ) encourages Th1 cellular immune responses, as well as Th2 cells, seem to need pro-inflammatory interleukins (such as IL-4 or IL-13) for their growth [11]. The conception that the immune system, which often successfully protects the host from microbial pathogens, might also recognize and destroy tumor cells was envisioned 50–100 years ago and is still under deep investigation. Over the past, several studies have been shown the immune system control over tumor formation and progression, through isolation and elimination of transformed cells, and suppression of in situ tumor-infiltrating lymphocytes or improving PRRs-PAMPs bind, and migration of antigen-presenting cells (APCs). Although a lot of attention has been given to innate immunity mechanisms, the activation of such proinflammatory effector T cells proposes a novel way to target the tumor microenvironment, potentially giving them substantial clinical value, especially in patients with glioblastoma. For instance, the high density of CD8+ cells at the site of the tumor is evocative of a specific immune response to antigens being T cell recognition and production of interferons (IFNs) correlated to an inducible PD-L1 expression as confirmed by T cells and PD-L1 colocation at interfaces of cancerous samples [12]. Another subset of T cells that utilize alpha–beta T-cell receptor (TCR) chains that are known to mediate target cell killing and instructive cytokine release are the natural killer (NK) T cells, granular lymphocytes secreting cytokines, and chemokines. As a result, the NK connection to their respective receptors highlights the bind to host or pathogens encoded ligands upregulation on ‘stressed’ or infected cells, starting hereinafter the adaptive immune cascade. Additionally, NK cell recruitment is directed by cellular integrated clues, including adhesion molecules and participation of chemotactic factors. It is important to mention that some mutations in glioblastoma, such as the upregulation of growth factor (GF) signaling pathways and/or the loss of cell cycle players, let glioblastoma cells override from immune control through a resistance mechanism to NK cells [13].

## Brain tumors progression and immune system control

Primary malignant central nervous system (CNS) tumors represent about 2% of all cancers but account for a disproportionate incidence of prevalence and mortality [14]. An estimated frequency of brain tumors is 14.8 per 100,000 person-years, with approximately half being histologically benign. CNS tumors are grouped pathologically, on the basis of the most malignant area identified, according to either the World Health Organization (WHO) system, which is based on the presence or absence of nuclear atypia, defined as a structural abnormality, defective mitosis, as DNA damage, microvascular proliferation, and necrosis. WHO classification specifies a grading system, ranging from grade I through grade IV. Astrocytoma is linked with a low-grade of fibrillary destruction (grade I/II) while, meningiomas and glioblastoma multiforme (GBM), refer to the most mutual malignant brain tumors (grade III/IV) [15]. Although all of these tumor types share a common tissue of residence, the brain, they are imperiled to various inputs from their own microenvironment due to variance in evolutionary history (e.g., primary brain tumors versus metastases). As tumor epidemiologists reported, specific types of adult brain tumors are associated with an umbrella of varied individual risk factors that can cross the blood–brain barrier (BBB) modifications and other brain route defects. For instance, risk factors for brain tumors include radiation exposure, hereditary syndromes and family history, exogenous hormones and, increasing age [16]. The CNS was once felt to be an immune-privileged area, that was because of the BBB, composed by the endothelial cells enclosed by astrocytes, which was believed to obstruct immune cells and mediators of immunity from reaching the CNS. Instead, multiples are the ways in which the CNS and the immune system both interact and interplay, as example, many cytokines are able to cross the BBB (i.e., IL-1, IL-6, and TNF-α) following injuries or founded neuroinflammatory pathologies [17]. Moreover, in support of neuroimmune-related events occurring into the CNS and over, rate of leukocytes entering the CNS is increased in the experimental autoimmune encephalomyelitis (EAE), a model of multiple sclerosis which appears associated to a high level of leukocyte infiltration into the CNS, as well as blood-derived immune cells infiltration into the CNS became possible due to a disrupted BBB seen in GBM microenvironment [18]. Accordingly, in patients harboring malignant gliomas, immune function results impaired, getting from these patients an exhibition of varied responses from B cell and T cell signaling defects, identified in an altered transmembrane signaling pathway, such as weakened ability to mobilize calcium (Ca) through Ca-activated potassium channels in glioma patients [19], reduced affinity to IL-2R expression/signaling and apoptosis of T cells through Fas ligand (FasL)-mediated activity. First, the BBB protects the brain, but it represents an obstacle for the diffusion of therapeutic molecules over the brain. BBB microvessels supporting brain tumors forming a blood–brain tumor barrier (BTB). Thus, because of KCa channels localization on metastatic tumor cells, the concept which encourages an adequate delivery of drugs can be sustain by a negative modulation of these channels’ activity [20]. Second, by the first report in 1985, a series of studies have been explained IL-2 application as cancer immunotherapy preventing metastatic renal cancer and metastatic melanoma [21]. Thus, enhancing IL-2 binding to the own receptors (IL-2Rβγ or IL-2Rαβγ) leads to the activation of multiple signaling pathways [22], involving three major downstream signaling pathways such as the STAT, the phosphoinositide 3-kinase (PI3K-AKT), and the mitogen-activated protein kinase (MAPK) signaling pathways, mainly in charge to mediate the cell growth, differentiation, and activation of T and NK cells [23]. Additionally, since IL-2 promotes the survival of memory CD8+ and CD4+ T cells, IL-2 treatment results in immune cell accumulation in tumors in a xenograft murine model of GBM [24]. Third, apoptosis of tumor-reactive T cells through Fas–FasL pathway has been a focus of study in relation to apoptosis on the basis of Fas/FasL system involvement in the control of immune homeostasis. Fas/FasL expression has recently been described in varied human malignancies, including hepatocellular carcinoma, lung cancer, colon carcinoma, and astrocytoma where its targeting may reduce tumor-infiltrating lymphocytes production [25]. Nowadays, modern research has turned to immunotherapies, using immune checkpoint inhibitors to modulate the undominated immune system especially in the presence of metastases [26]. Brain metastases (BM) are devastating complications occurring in up to 40% of cancer patients with cancer from various neoplasms, mainly from lung cancer, breast cancer, and melanoma. Particularly, the TME where BM had spread can includes cancerous and noncancerous cells including endothelial cells, pericytes, fibroblasts, and last but not least immune cells. Immune scenario escape is an emerging hallmark of cancer since BM initiating cancer cells have to evade immune various attacks throughout the brain metastatic cascade. However, besides immune escape, it has been postulated that immune cells possess supporting functions in the process of metastatic spread and are used by the cancer cell to support their growth [27]. Markers for response to immune checkpoint inhibition include relatively high tumor mutation burdens, elevated expression of immune checkpoints such as tumoral programed cell death ligand 1 (PD-L1) and a ‘hot’ or inflamed state characterized by tumor-infiltrating lymphocytes (TILs) [28].

## PD1/PDL-1 pathway mediating brain tumor progression and escape

As mentioned above, lately, the PD-1 role in cancer research as responsible for tumor immune modulation has attracted substantial interest. Belonging to the CD28 family, PD-1 (CD279) is one of the T cell co-inhibitory receptors, expressing on immune cells, such as activated T cells, regulatory T cells, natural killer cells (NK cells), activated B cells, and macrophages [29]. PD-1 has two recognized ligands (PD-L), PD-L1(B7-H1/CD274), primarily expressed on immune cells (T and B cells, DCs and macrophages) and non-immune cells, and PD-L2 (PD-L2/CD273), more restricted to immune cells. Expression of PD on tumors plays an important role in immune evasion due to the interaction of PD-L1 with the inhibitory receptor PD-1 that is expressed on activated lymphocytes and it is up-regulated in TMEs of different types of cancer [30]. Since it is well established that PDL-1 overexpression is significant in glioma samples, emerged data by using different glioma cell lines, murine and human samples, support the negative survival signal of PD-1/PD-L1 pathway may represent immune suppression mechanism employed by malignant brain tumors. Thus, the blockage of PD1/PDL-1 interaction exhibits strong cytotoxicity toward glioma cell lines and crosses the BBB, respecting physiological cellular proliferation, especially of immune cells [31]. Targeted PD-L1/PD-1 immunotherapy has not achieved the desired effects in the treatment of various types of cancers, especially for solid tumors, even because exact PD-1 localization is not clear. PD1/PD-L1 expression in all the glioma patients was weak or moderate positive, and PD-L1 expression was predominantly displayed on the cytoplasm and rarely presented in the cellular membrane, offering a specific response to immune activity in the TME even in non-uniform PD-L1 tumor cells [32]. Up to now, PD-L1/PD-1 immune cell interaction has been defined as canonical signaling, characterized by high infiltration of CD8+ T cells and conventional CD4+ T cells, while recent studies found that PD1 non-canonical via, inhibits proliferation of cancer cell via suppressing AKT and ERK signaling hence it plays an important role in tumorigenesis, suggesting that should be a potential therapeutic strategy. PD1/PDL-1 has been observed not only on glioma but even on astrocytoma cell lines up to reduces IFN-γ production of allogeneic T cells and improves neuronal cell long-term survival in mice with intracranial gliomas [33]. Although studies on PD-1 and glioma in humans remain extremely limited, preclinical evidence for PD-1’s inhibitory role comes from several fronts. Among different histological types of brain tumor, astrocytoma (WHO grade II-IV), oligodendrogliomas (WHO grade II-III), ependymomas (WHO grade II-III), and pilocytic astrocytoma (WHO grade I), in which the most malignant GMB (grade IV) belongs to astrocytoma, PD-1 cascade, especially in CD4+ T cells, reflect the severity of the disease is clearly increased in grade III (12.3 ± 0.9%) and IV patients (16.3 ± 0.6%), with the grade IV having the highest value [34]. While immunotherapy advances reflecting potential possibilities for meningiomas treatment, very little is known of their TME, especially regarding higher-grade meningiomas, in which radiotherapy is commonly applied only as an adjuvant treatment. Based on Han S. and colleagues experience, PD/PD-L1 significance in meningiomas is consistent as showed by CD68+ double staining of tumor-infiltrating cells in a significant subset of meningiomas cases proposing PD-L1 via could play a biologic role in the hostility for grade II/III and radiotherapy failed meningiomas [3].

## PD-1/PDL1 blockage interaction as brain tumor immunotherapy

The last advances of immune checkpoint inhibitors have reversed the treatment archetype for many tumor types. Since PD-L1 is an essential immune checkpoint protein that binds to PD-1 on T cells and plays a critical role in killing cancer cells, attenuating the occurrence of immune activation in the TME, or inhibiting the release of co-molecules and pathways involved may be a desirable effect for tumor treatment. When PD-1 binds to PD-L1, it basically says the T cell “to leave the other cell alone”. Some cancer cells have large content of PD-L1, which helps them hide from an immune outbreak. For example, monoclonal antibodies that target either PD-1 or PD-L1 can block this binding and boost the immune response against cancer cells. A large variety of drugs have shown a promised use in treating definite cancers. Since the approval of pembrolizumab, (brand name: Keytruda, also known as MK-3475 or lambrolizumab) a humanized IgG4 kappa anti-PD-1 antibody with a high affinity for the treatment of advanced melanoma, the clinical development of PD-1 and PD-L1 inhibitors as anticancer agents has expanded. Indeed, pembrolizumab showed good tolerability in phase I study that involved solid tumor patients associated with durable antitumor activity [35]. Although the promising choice of pembrolizumab for GBM treatment, its use as monotherapy has been reported to possess limited efficacy compared with control patients [36].

**Fig. 2:**
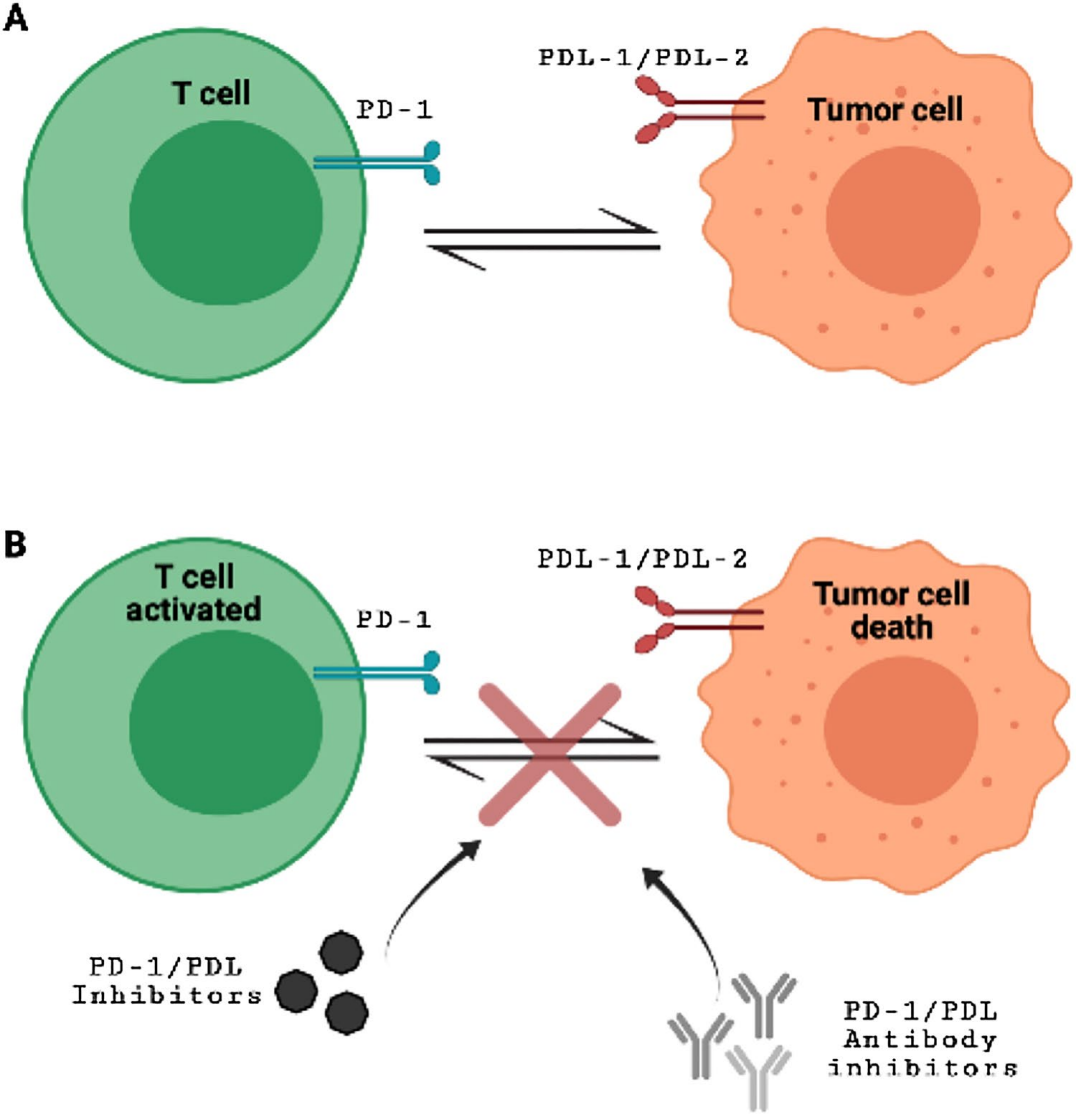
PD-1/PDL-1 immune checkpoint in tumorigenesis (Panel **A**). PD-1/PDL-1 blockade by targeted antibodies and specific inhibitors (Panel **B**)

A previous meta-analysis stated that PD-1/PD-L1 inhibitors are better tolerated than predictable chemical and radioactive therapies, and such evidence in the literature regarding immunotherapeutic treatment with immune-checkpoint inhibitors in patients with GBM is taking place and are reporting use of monotherapy or a combination of PD/PDL-1 inhibitors with other drugs [37]. Presently, the FDA has approved PD-1/PD-L1 inhibitors for the treatment of 9 cancer types (https://www.fda.gov/drugs/resources-information-approved-drugs/hematologyoncology-cancer-approvals-safety-notifications) with immune checkpoint inhibitor monotherapy. A various number of immune checkpoint blockers are currently under investigation alone or in combination with PD-1 block. Firstly, a randomized phase I/II study has been scheduled for pidilizuma (known also as Yervoy), a T cells specific monoclonal antibody, already available for advanced melanoma treatment and recently under investigation for glioma and GBM (NCT01952769) (CRUK internal database number: 16025) (https://www.cancerresearchuk.org/about-cancer/find-a-clinical-trial/a-trial-of-ipilimumab-andtemozolomide-for-people-with-glioblastoma-ipi-glio#undefined). In December 2014, the FDA accelerated the approval of nivolumab, (brand name: Opdivo, also known as BMS-936558 and MDX1106) a humanized IgG4 anti-PD-1 monoclonal antibody that has a high affinity for PD-1, blocking the binding of PD-1 to its ligand PD-L1 (Fig. 2), for treating metastatic melanoma [38]. Of great importance, nivolumab showed a constant and safety profile also in GBM patients compared to other tumor types [39] but there is still a lot to be found out about the use of Nivolumab in the resolution of GBM progression. Currently, Nivolumab is examined as a "neoadjuvant" treatment in a clinical-stage II (NCT 02,550,249) for patients with recurrent GBM which urges surgical removal. Right here, at phase II of a clinical trial, Durvalumabis (MEDI4736 also known as Durva), a human IgG1 monoclonal antibody that blocks PD/PD-L1 immune checkpoint, is recommended by FDA for a restricted cohort of patients with bladder and lung cancers. Therefore, it showed the best responses in 13.3% and stable disease in 46.7%, falling adverse events within 12 months. In 2019, a well-detailed clinical study reported high safety and efficacy of Durvalumabis associated with radiotherapy (RT) in GBM patients. Durvalumabis + RT association was well tolerated seeming to have efficacy among patients with new unmethylated GBM. Moreover, some specific drugs targeting the PD-L1/PD-1 pathway act through PD-L1 protein cell processing influence. PD-L1 protein degradation that occurs in proteasomes or lysosomes could use multiple pathways of degradation, leading to enriched immunotherapy strategies for cancer, and providing a potential strategy to increase the response rates of PD-1/PD-L1 blockade in cancer immunotherapy [40]. In this context, overactivation of ubiquitinoylation-related pathways such as neddylation, a post-translational modification process by which the ubiquitin-like protein NEDD8 is conjugated to its target proteins, and the simultaneous inhibition of PD-L1/PD-1 checkpoint by combined therapy, may be benefit in GBM patients by successful reverse of T cells maturation [41, 42]. Since PD-L1/PD-1 immune checkpoint blockade has transformed the treatment of many different cancers’ types and states, accumulating data also sustenance the basic concept that corticosteroid use at the same time of immune checkpoint therapy may give concerns. As a matter of facts, it has been reported that anti-PD-L1 treatment associated with corticosteroids limits the therapeutic effects of immune checkpoint modulation by poorer survival in GBM patients [43].

## Future perspectives

Lately, progresses in cancer immunotherapy, with the gain of checkpoint blockade have caused a worldview to move in cancer progression. After numerous investigations, the fundamental components behind tumor evasion have been explained which led to the improvement of successful immunotherapeutic techniques. Moreover, earlier clinical trials, have been reported these advancements but including a minority of patients. In terms to address these issues, the goal of immunotherapy research requires the selection of the patients that will advantage the foremost from particular immunotherapeutic modalities, with the utilize of predicted biomarkers. Biomarkers that can be detected in peripheral blood from brain such as the intercellular cell adhesion molecule-1 (ICAM-1) or human leukocyte antigen-antigen D related (HLA-DR), could represent a significant approach in the research of tumor immune microenvironment as well to verify the response to any anti-PD-1/PD-L1 treatments in GBM patients. For example, when using anti-PD-1 or PD-L1 antibodies to treat different types of cancer, some patients with a low PD-L1 expression might be inadequate responders because of differences in PD-L1 expression levels among patients, PD-L1 functionality, or inappropriate tissue immunohistochemistry (IHC) technique. Therefore, further studies could investigate the mechanisms of PD1/PDL-1 checkpoint in combination with another immune checkpoint (i.e. the cytotoxic T lymphocyte-associated antigen 4 or CTLA-4) for targeting a variety of tumors including brain tumor.

## Conclusions

As a matter of fact, that cancer immunotherapy focusing on PD-1 or PD-L1 has demonstrated exciting reactions in causing strong antitumor responses. We accept that PD-1/PD-L1 barricade treatment will be the major cancer immunotherapy strategy. Although, phase I and phase II trials of PD-1/PD-L1 inhibitors, have reported promising efficacy outcomes, new approaches in tumor immunobiology are needed.

## Data Availability

Not applicable.
